# Uromodulin Retention in Thick Ascending Limb of Henle's Loop Affects SCD1 in Neighboring Proximal Tubule: Renal Transcriptome Studies in Mouse Models of Uromodulin-Associated Kidney Disease

**DOI:** 10.1371/journal.pone.0113125

**Published:** 2014-11-19

**Authors:** Marion Horsch, Johannes Beckers, Helmut Fuchs, Valérie Gailus-Durner, Martin Hrabě de Angelis, Birgit Rathkolb, Eckhard Wolf, Bernhard Aigner, Elisabeth Kemter

**Affiliations:** 1 German Mouse Clinic, Institute of Experimental Genetics, Helmholtz Zentrum München GmbH, German Research Center for Environmental Health, Neuherberg, Germany; 2 German Center for Diabetes Research (DZD), Neuherberg, Germany; 3 Experimental Genetics, Center of Life and Food Sciences Weihenstephan, Technische Universität München, Freising-Weihenstephan, Germany; 4 German Center for Vertigo and Balance Disorders, University Hospital Munich, Campus Grosshadern, Munich, Germany; 5 Molecular Animal Breeding and Biotechnology, and Laboratory for Functional Genome Analysis (LAFUGA), Gene Center, LMU München, Munich, Germany; University of Maryland School of Medicine, United States of America

## Abstract

Uromodulin-associated kidney disease (UAKD) is a hereditary progressive renal disease which can lead to renal failure and requires renal replacement therapy. UAKD belongs to the endoplasmic reticulum storage diseases due to maturation defect of mutant uromodulin and its retention in the enlarged endoplasmic reticulum in the cells of the thick ascending limb of Henle's loop (TALH). Dysfunction of TALH represents the key pathogenic mechanism of UAKD causing the clinical symptoms of this disease. However, the molecular alterations underlying UAKD are not well understood. In this study, transcriptome profiling of whole kidneys of two mouse models of UAKD, *Umod* ^A227T^ and *Umod* ^C93F^, was performed. Genes differentially abundant in UAKD affected kidneys of both *Umod* mutant lines at different disease stages were identified and verified by RT-qPCR. Additionally, differential protein abundances of SCD1 and ANGPTL7 were validated by immunohistochemistry and Western blot analysis. ANGPTL7 expression was down-regulated in TALH cells of *Umod* mutant mice which is the site of the mutant uromodulin maturation defect. SCD1 was expressed selectively in the S3 segment of proximal tubule cells, and SCD1 abundance was increased in UAKD affected kidneys. This finding demonstrates that a cross talk between two functionally distinct tubular segments of the kidney, the TALH segment and the S3 segment of proximal tubule, exists.

## Introduction

Uromodulin-associated kidney disease (UAKD) is a rare dominant hereditary renal disease caused by amino acid-changing mutations in the uromodulin (*UMOD*) gene [1]–[3]. Patients with UAKD exhibit impaired urinary concentration ability, in most cases hyperuricemia, morphological kidney alterations like progressive tubulointerstitial damage and sometimes renal cysts and constantly develop disease progression up to renal failure. Dysfunction of thick ascending limb of Henle's loop (TALH) cells due to mutant UMOD maturation retardation and retention in the hyperplastic endoplasmic reticulum (ER) represents the key pathogenic mechanism of UAKD.

Uromodulin is selectively expressed in cells of the TALH and of the early distal convoluted tubules [4]. After UMOD synthesis in the ER and extensive glycosylation, the mature protein is translocated to the luminal cell membrane and released into urine by proteolytic cleavage. UMOD represents the most abundant protein in human urine. Although this glycoprotein was already discovered in the early fifties by Tamm and Horsfall (therefore initially named Tamm-Horsfall-glycoprotein) [5], the biological function of UMOD is still obscure. Studies on *Umod* knockout mice revealed a protective role of UMOD against ascending urinary tract infections e.g. of type 1-fimbriated *E. coli*, and a protective role against calcium oxalate crystal formation in the kidney [6]–[8]. Further, UMOD might have a role in innate immune system and might act as an endogenous danger signal after tubular damage that leads to exposition of UMOD protein to mononuclear cells like dendritic cells in the kidney interstitium [9]. In various genome-wide association studies, common allelic *UMOD* promoter variants were identified to be associated with increased risk for complex trait diseases like chronic kidney disease (CKD), hypertension and kidney stones (reviewed in [3]). These variants of the *UMOD* promoter lead to increased UMOD expression and secretion which results, by influencing salt reabsorption in the kidney, to increased risk of developing hypertension and CKD [10].

UMOD maturation defect and retention in TALH cells in UAKD lead to unfolded protein response and activation of non-canonical NF-κB signaling in the TALH segment as demonstrated recently in two mouse models of UAKD, the mutant mouse lines *Umod* ^C93F^ and *Umod* ^A227T^ [11]. The focus of this study is to analyze transcriptional alterations in UAKD affected kidneys to obtain further insights into the pathogenesis of UAKD and/or the biological function of UMOD.

## Materials and Methods

### Animals

Both *Umod* ^A227T^ and *Umod* ^C93F^ mutant mouse lines, generated by ENU mutagenesis, exhibit the key features of UAKD like maturation defect and retention of UMOD in the hyperplastic ER of TALH cells and impaired kidney function with mild defect in urinary concentration ability, reduced fractional excretion of uric acid, and reduced UMOD excretion [12], [13]. *Umod* ^A227T^ and *Umod* ^C93F^ mutant mice differ in onset, severity and speed of progression of disease symptoms as these are dependent on kind of *Umod* mutation and allelic status [13].

Homozygous *Slc12a1* ^I299T^ mutant mice exhibit key features of Type I Bartter syndrome with salt-wasting polyuria and reduced fractional excretion of uric acid due to impaired ion transport activity of the main ion transporter of the TALH segment, the Na^+^-K^+^-2Cl^—^ion transporter NKCC2, which is the protein derived from the *Slc12a1* gene [14].

All three mouse lines were maintained on the C3HeB/FeJ (C3H) genetic background. Mouse husbandry was done under a continuously controlled specific pathogen-free (SPF) hygiene standard according to the FELASA recommendations (http://www.felasa.eu) [15]. Mouse husbandry and all tests were carried out under the approval of the responsible animal welfare authority (Regierung von Oberbayern, Germany).

### Genome-wide transcriptome analysis of UAKD-affected kidneys

The genome-wide transcriptome analyses of whole kidney lysates of *Umod* mutant mice were part of the systemic, comprehensive phenotypic analysis carried out in the German Mouse Clinic at the Helmholtz Zentrum München using standardized examination protocols (http://www.mouseclinic.de) [16], [17]. First, the analysis of kidneys of four male homozygous *Umod* ^A227T^ mutant mice and four male wild-type littermates was carried out at an age of 17 weeks, followed by the analysis of kidneys of four male heterozygous *Umod* ^C93F^ mutants and their wild-type littermates at an age of 38 weeks.

Total RNA of whole kidney was isolated using Trizol (Invitrogen, Karlsruhe, Germany) in combination with RNeasy Midi Kits according to manufacturer's protocol (Qiagen, Hilden, Germany). cDNA microarrays covering 17,346 mouse genes were in-house produced, dual color hybridization was performed and arrays were scanned as previously described [18]. As a first step of the expression profiling data from kidney low-quality array elements were eliminated applying several filter methods (background checking for both channels with a signal/noise threshold of 2.0, one bad tolerance policy parameter and flip dye consistency checking). In total, 9,237 useable expression values remained and were used for the identification of genes with significant differential gene regulation using SAM (Significant analysis of microarrays [18]–[20]). Genes were ranked according to the mean fold change (mean mutant divided by mean wild-type signal intensity) and selected as significantly differentially expressed with ratio >1.4 in combination with false discovery rate (FDR) <10%. The FDR is a value which estimates the percentage of nonsense genes by calculating 1000 permutations.

Over-represented functional annotations within the data sets were provided as GO (Gene Ontology terms of the category ‘functions and diseases’ (Ingenuity Pathway Analysis, IPA)). IPA was also used to identify biological associations based on co-citations and co-expression between the regulated genes of each of the two mutant lines and the NF-κB signaling complex. In the GEO database [21], the complete microarray dataset is available under accession number GSE58513.

### Quantitative real time PCR analysis

After isolation of total RNA from whole kidneys of four-month-old male homozygous *Umod* mutant mice, three-month-old male homozygous *Slc12a1* ^I299T^ and corresponding age-matched male wild-type littermates with the Trizol method, cDNA synthesis was performed using cDNA EcoDry Premix Double Primed (Clontech). NCBI/Primer-BLAST (http://www.ncbi.nlm.nih.gov/tools/primer-blast/index.cgi?LINK_LOC=BlastHome) was used to select cDNA specific primers from different exons (except for *Hba-a1*) and each primer pair was tested for comparability of amplification efficiencies by performance of real-time PCR-based standard curve analyses. Primer sequences are listed in Table 1. Transcript abundance was quantified using FastStart Universal SYBR Green Master (ROX) (Roche) on a StepOne Real-Time PCR system (Applied Biosystems) and using LinRegPCR software [22]. All real-time PCR measurements were performed in duplicates and included no template controls. Transcript abundances of *Angptl7*, *Hba-a1*, *Odc1*, *Scd1*, and *Wfdc15b* were calculated in relation to the expression of the housekeeping genes *Sdha*, *Tbp*, and *Rpl13a* using the 2^−ΔCT^ method.

**Table 1:** List of primer sequences used for RT-qPCR.

Gene	Sequence (5′-3′)		Length	Accession No.
*Angptl7*	forward: TCCGAAAAGGTGGCTACTGG	reverse: ATGCCATCCATGTGCTTTCG	97 nt	NM_001039554.3
*Hba-a1*	forward: GTGCATGCCTCTCTGGACA	reverse: GGTACAGGTGCAAGGGAGAG	127 nt	NM_008218.2
*Odc1*	forward: CCGGCTCTGACGATGAAGAT	reverse: CTTCTCGTCTGGCTTGGGTC	146 nt	NM_013614.2
*Rpl13a*	forward: GACCTCCTCCTTTCCCAGGC	reverse: GCCTCGGCCATCCAATACC	70 nt	NM_009438.5
*Scd1*	forward: AGGCCTGTACGGGATCATACT	reverse: AGAGCGCTGGTCATGTAGTAG	84 nt	NM_009127.4
*Sdha*	forward: AACACTGGAGGAAGCACACC	reverse: AGTAGGAGCGGATAGCAGGA	135 nt	NM_023281.1
*Tbp*	forward: TCTGGAATTGTACCGCAGCTT	reverse: ATGATGACTGCAGCAAATCGC	131 nt	NM_013684.3
*Wfdc15b*	forward: TTTCGCATACGGAGGACAGT	reverse: GGGCCAGGTGTGGTTATGTC	79 nt	NM_001045554.1

cDNA-specific primers for amplification of mouse angiopoietin-like 7 (*Angptl7*), hemoglobin alpha adult chain 1 (*Hba-a1*), ornithine decarboxylase structural 1 (*Odc1*), stearoyl-Coenzyme A desaturase 1 (*Scd1*), WAP four-disulfide core domain 15B (*Wfdc15b*), and the housekeeping genes ribosomal protein L13A (*Rpl13a*), succinate dehydrogenase complex subunit A flavoprotein (*Sdha*) and TATA box binding protein (*Tbp*).

### Immunohistochemical analyses

Histological analyses of kidneys of homozygous *Umod* mutant mice and wild-type mice were performed as described previously [12]. Immunohistochemistry was performed using the following primary antibodies: rat monoclonal antibody against mouse ANGPTL7 (clone 538401; R&D Systems), rabbit monoclonal antibody against mouse SCD1 (#2794, Cell Signaling), and rat monoclonal antibody against mouse UMOD (clone 774056; R&D Systems). Immunoreactivity was visualized using 3,3-diaminobenzidine tetrahydrochloride dihydrate (DAB) (brown color) or using Vector Red Alkaline Phosphatase Substrate Kit I (red color). Nuclear counterstaining was done with hemalum. Cells of TALH segment were identified by UMOD immunostaining. Cells of proximal tubule segment were identified by morphological criteria of presence of luminal microvilli.

### Western blot analyses

Renal tissue (whole kidney or outer medullar region) of homozygous *Umod* mutant mice and wild-type mice was homogenized in Laemmli extraction buffer (20 mM Tris, 2% Triton-X100, 20% 5× Laemmli buffer). Outer medullar region contained a higher fraction of TALH segments and was prepared as described previously [11]. Protein concentration was determined by BCA assay. Equal amounts of denatured proteins per lane were separated on 12% SDS-polyacrylamide minigels and blotted on PVDF membranes. Equal loading was controlled by Ponceau staining.

The following primary antibodies were used: rat monoclonal antibody against mouse ANGPTL7 (clone 538401; R&D Systems), rabbit monoclonal antibody against GAPDH (#2118, Cell Signaling), rabbit polyclonal antibody against mouse SCD1 (#2438, Cell Signaling). Bound antibodies were visualized using ECL reagent (GE Healthcare Amersham Biosciences). Signal intensities were quantified using ImageQuant (GE Healthcare). Standardization of equal loading was referred to the signal intensities of GAPDH of the corresponding PVDF membrane.

### Statistical analysis

Statistical analyses of genome-wide transcriptome analyses were described above. Statistical analyses of data derived by RT-qPCR analyses and Western blot analyses were carried out by One-way ANOVA with Tukey's Multiple Comparison Post hoc Test if comparing three groups, and by unpaired Student's *t*-test if comparing two groups. Statistical analyses of plasma urea data were performed by Two-way ANOVA with Bonferroni Multiple Comparison Post hoc Test. Data are shown as mean ± standard deviations. Significant differences are indicated for *P*<0.05, 0.01, and 0.001.

## Results

### Transcriptome profiling of UAKD-affected kidneys

The primary phenotype reports of both mutant lines *Umod^A227T^* (see line “HST012”) and *Umod^C93F^* (see line “HST001”) of the German Mouse Clinic are accessible online (http://146.107.35.38/phenomap/jsp/annotation/public/phenomap.jsf) [17]. For the following analysis, the kidney transcriptome data of both *Umod* mutant lines were analyzed using enhanced settings in the software programs (see Materials and Methods). For genome-wide transcriptome profiling of whole kidneys, one group of UAKD-affected mice with initial mild disease phenotype, the young-adult homozygous *Umod* ^A227T^ mutants, and another group with progressed disease state of UAKD including morphological kidney alterations, the aged heterozygous *Umod* ^C93F^ mutant mice, were used (Figure S1).

First, genome-wide transcriptome profiling analysis of kidneys from homozygous *Umod* ^A227T^ mutant mice, carried out at an age of 17 weeks, revealed 104 differentially expressed genes (DEGs) compared to age-matched wild-type littermate controls (Figure S2). In order to identify over-represented functional annotations among the set of regulated genes, Ingenuity Pathway Analysis (IPA) was employed. In kidney of *Umod* ^A227T^ mutants, the DEGs were associated with proliferation of cells, necrosis, inflammation, metabolism of lipid and proteins as well as renal cancer (Table 2, Figure S4). Several up-regulated genes are known to be expressed in proximal and/or distal tubules like *Abcc4*, *Atp1a1*, *Atoh8*, *Clcnkb*, *Col18a1*, *Car15* and *Mt1* and are required for normal reabsorption and urine concentration. *Fabp4*, *Gsn*, *Il6st*, and *Ly6e*, were associated with nephritis and *Col18a1*, *Prom1*, and *Scd1* with renal cell carcinoma. Further, several differential abundant genes were associated with repression or activation of NF-κB signaling complex (*Atp1a1*, *Cxcl12*, *Fabp4*, *Hbb*, *Itgav*, *Mt1*, *Scd1*, *Serpina1*, and *Tacstd2*). The other way round, the NF-κB signaling complex plays roles in the expression of *Cxcl12*, *Fabp4*, *Fgf1*, *Gclc*, *Odc1*, and *Tap*.

**Table 2:** Functional classification of differentially expressed genes in kidney of *Umod* ^A227T^ mutant line.

Categories	Diseases or Functions Annotation	p-Value	Genes	# Genes
Cancer	abdominal neoplasm	6.37E-03	*Abcc4, Acsl5, Actg1, Ahnak, Aldoa, Anxa5, Atp1a1, Col18a1, Cpa1, Cxcl12, Dnajc10, Dpp10, Egr1, Eno1, Fabp4, Fgf1, Grin2a, Gsn, Hba1/Hba2, Hbb, Il6st, Itgav, Ivns1abp, Ly6e, Mapre1, Mark3, Mt1, Nup155, Odc1, Pcolce, Prom1, Rbm3, Rimbp2, Rps4y1, S100a11, Sat1, Scd, Serpina1, Tacstd2, Tap1, Wfdc2*	41
Cellular Growth and Proliferation	proliferation of cells	1.47E-05	*Abcc4, Acsl5, Actg1, Ahnak, Aldoa, Cd24a, Col18a1, Cxcl12, Dnph1, Eef1a1, Egr1, Eno1, Fabp4, Fgf1, Gclc, Grin2a, Gsn, Hba1/Hba2, Igkv1-117, Il6st, Itgav, Ivns1abp, Mapre1, Morf4l1, Mt1, Odc1, Rbm3, Rps14, S100a11, Sat1, Scd, Serpina1, Tacstd2, Tap1*	34
Cell Death and Survival	necrosis	3.36E-06	*Abcc4, Aldoa, Ap2b1, Atp1a1, Cd24a, Col18a1, Cxcl12, Eef1a1, Egr1, Eno1, Fabp4, Fbxo32, Fgf1, Gclc, Grin2a, Gsn, Gsta1, Igkv1-117, Il6st, Itgav, Ivns1abp, Mt1, Odc1, Rbm3, S100a11, Sat1, Scd, Serpina1, Ucp1*	29
Inflammatory Response	inflammation of organ	1.11E-07	*Abcc4, Ahnak, Aldoa, Anxa5, Atp1a1, Cxcl12, Eef1a1, Egr1, Eno1, Fabp4, Fbxo32, Gsn, H3f3a/H3f3b, Hbb, Il6st, Mt1, Odc1, Prom1, S100a11, Scd*	20
Molecular Transport	transport of molecule	6.05E-05	*Abcc4, Acsl5, Atp1a1, Clcnka, Cxcl12, Egr1, Fabp4, Fgf1, Grin2a, Gsn, Hba1/Hba2, Hbb, Il6st, Itgav, Ly6e, Nup155, Scd, Tap1, Ucp1*	19
Lipid Metabolism	synthesis of lipid	4.20E-05	*Abcc4, Acsl5, Atp1a1, Cxcl12, Eef1a1, Egr1, Fgf1, Gsta3, Hbb, Odc1, Scd, Serpina1, Ucp1*	13
Protein Synthesis	metabolism of protein	7.61E-04	*Cpa1, Eef1a1, Fbxo32, Grin2a, Gsn, Pcolce, Rbm3, Rps14, Rps4y1, Sat1, Serpina1, Wfdc2*	12
Metabolic Disease	diabetes mellitus	6.97E-03	*Anxa5, Fabp4, Fgf1, Grin2a, Hba1/Hba2, Hbb, Itgav, Mt1, Nup155, Scd, Tap1*	11
Carbohydrate Metabolism	quantity of carbohydrate	6.48E-04	*Anxa5, Eef1a1, Fabp4, Fgf1, Il6st, Mark3, Mt1, Scd, Ucp1*	9
Cardiovascular Disease	hypertension	1.97E-03	*Atp1a1, Clcnka, Cpa1, Fbxo32, Fgf1, Gsn, Gsta3, Hba1/Hba2, Il6st*	9
Renal Disease	renal cancer	4.83E-03	*Ahnak, Anxa5, Gsn, Mt1, Nup155, S100a11, Scd, Tacstd2*	8
Cell-To-Cell Signaling	binding of cells	7.10E-03	*Anxa5, Cd24a, Col18a1, Cxcl12, Fgf1, Gsn, Itgav*	7

Second, 54 DEGs were detected in whole kidney lysate of 38-week-old heterozygous *Umod* ^C93F^ mutants when compared to age-matched wild-type littermate controls (Figure S3). Necrosis, inflammation, lipid metabolism, hypertension and distinct renal and urological functions/disease were over-represented GO terms in this dataset (Table 3, Figure S5). Expression in the proximal and/or distal renal tubules was described for *Kap*, *Lgmn*, *Pdzk1*, *Slc13a1*, and *Slco4c1*, which were down-regulated in kidneys of *Umod^C93F^* mutants. Further genes were annotated with nephritis (*Cndp2*, *Dnase1* and *Scd1*). While *Timp1* and *Odc1* expression depends on the activity of the NF-κB signaling complex, *Akr1b1, Casp9, Mt2* and *Scd1* diminish the activity of the transcription factor complex.

**Table 3:** Functional classification of differentially expressed genes in kidney of *Umod* ^C93F^mutant line.

Categories	Diseases or Functions Annotation	p-Value	Genes	# genes
Cancer	cancer	5.85E-03	*Acox1, Acsm2a, Agps, Akr1b1, Ank1, C11orf54, Casp9, Cchcr1, Cndp2, Cyp51a1, Dnase1, Gpm6a, Grm2, Hba1/Hba2, Hgd, Hsd17b11, Il5ra, Inmt, Lgmn, Macrod1, Mt2, Odc1, Pah, Pank1, Pank3, Pdzk1, Rtn3, Scd, Slc13a3, Snx27, Timp1, Tmem174, Ttr, Ube2a, Ugt2b28, Ugt2b7*	36
Cell Death and Survival	necrosis	5.17E-03	*Akr1b1, Casp9, Cchcr1, Dnase1, Gpm6a, Grm2, Il5ra, Kap, Lgmn, Mt2, Odc1, Pdzk1, Scd, Timp1, Ttr*	15
Molecular Transport	transport of molecule	1.00E-02	*Ank1, Casp9, Cchcr1, Grm2, Hba1/Hba2, Pdzk1, Scd, Slc13a3, Snx27, Ttr*	10
Inflammatory Response	inflammation	3.68E-03	*Aco2, Acox1, Dnase1, Il5ra, Lgmn, Mt2, Odc1, Scd, Timp1*	9
Lipid Metabolism	concentration of lipid	5.51E-04	*Agps, Akr1b1, Casp9, Kap, Pank1, Pdzk1, Scd, Timp1, Ttr*	9
Cardiovascular Disease	hypertension	6.97E-03	*Acox1, Cyb5b, Hba1/Hba2, Inmt, Kap, Pah*	6
Protein Synthesis	quantity of protein in blood	1.94E-03	*Hba1/Hba2, Lgmn, Mt2, Pdzk1, Scd, Ttr*	6
Renal Disease	renal cancer	1.99E-02	*Cyp51a1, Gpm6a, Mt2, Scd, Timp1*	5
	failure of kidney	1.98E-04	*Dnase1, Hba1/Hba2, Lgmn, Mt2, Ttr*	5
	cell death of renal tubule	4.90E-04	*Dnase1, Kap, Mt2*	3
	proliferation of kidney cell lines	6.54E-03	*Cchcr1, Mt2, Ttr*	3
	nephritis	2.41E-02	*Dnase1, Il5ra, Mt2*	3
	abnormal morphology of renal tubule	1.57E-03	*Akr1b1, Lgmn, Mt2*	3
	end stage disease	1.96E-03	*Lgmn, Mt2, Ttr*	3
	glomerulosclerosis	3.08E-03	*Kap, Lgmn, Mt2*	3
	urination disorder	3.84E-02	*Akr1b1, Kap, Lgmn*	3
Post-Translational Modification	hydrolysis of protein fragment	2.69E-02	*Casp9, Cndp2, Lgmn*	3

To identify DEGs irrespective of secondary morphological kidney alterations due to different UAKD disease progression stage, transcriptome datasets of the *Umod* ^A227T^ mutant mouse line were compared with that of the *Umod* ^C93F^ mutant mouse line. Hierarchical cluster analysis of all differentially expressed genes in both mutant lines displayed a similar tendency of transcriptional alteration for about 43% of the genes identified as DEGs in one of both *Umod* mutant mouse lines but which were only significantly regulated in one of the two lines (data not shown). An overlap of 5 DEGs (*Angptl7*, *Hba-a1*, *Odc1*, *Scd1* and *Wfdc15b*) was identified between the datasets of regulated genes of both *Umod* mutant lines. *Scd1* was overexpressed and *Angptl7*, *Hba-a1*, *Odc1* and *Wfdc15b* displayed significant down-regulation in *Umod^A227T^* and *Umod^C93F^*.

### Validation of microarray datasets by quantitative RT-real time PCR

**Figure 1:**
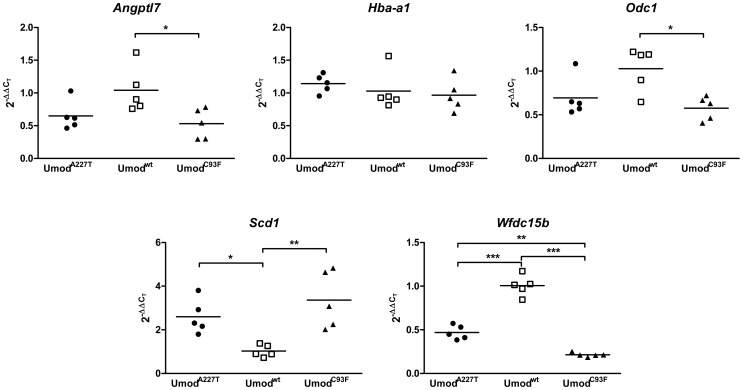
Verification of DEGs, identified by transcriptome profiling of whole kidneys, by RT-qPCR. Data are shown as scatter dot plot with mean (n = 5 per group). Age of mice analyzed: four months. One-way-ANOVA with Tukey's Multiple Comparison Post hoc Test: *p* vs. wild-type, *, *p*<0.05; **, *p*<0.01; ***, p<0.001.

Genes identified as equally differentially abundant in both *Umod* mutant mouse lines by genome-wide transcriptome analyses were selected for quantitative RT-real time PCR (RT-qPCR) analyses of whole kidney lysates of four-month-old male mice (n = 5 per group) (Figure 1). *Scd1* transcripts were significantly higher abundant in kidneys of both homozygous *Umod* ^C93F^ and *Umod* ^A227T^ mutant mice compared to wild-type mice by RT-qPCR analyses, whereas *Wfdc15b* exhibited a significantly decreased transcript abundance in both *Umod* mutant mouse lines. *Angptl7* and *Odc1* exhibited significantly decreased transcript abundances in whole kidney lysates of homozygous *Umod* ^C93F^ mutant mice and showed the tendency of decreased transcript abundances also in homozygous mutants of the *Umod* ^A227T^ mouse line which exhibited a less severe UAKD phenotype and TALH dysfunction compared to age-matched mutant mice of the *Umod* ^C93F^ mouse line [13]. RT-qPCR of *Hba-a1* revealed similar transcript abundances irrespective of genotype. In summary, RT-qPCR analyses of independent groups of mice confirmed the results for *Angptl7*, *Odc1*, *Wfdc15b* and *Scd1*, obtained by array hybridization, and differed only for *Hba-a1*.

### Localization and quantification of ANGPTL7 and SCD1 in the kidney by immunohistochemistry and Western blot analysis

**Figure 2:**
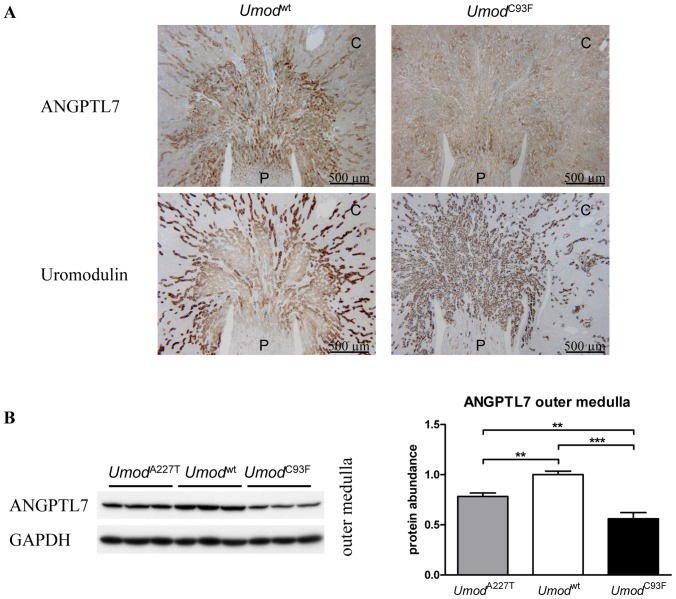
Analysis of localization and protein abundance of ANGPTL7 in healthy and UAKD-affected kidneys. (**A**) ANGPTL7 was predominantly detected in the cytoplasmic compartment of tubular cells, predominantly in TALH cells, of wild-type mice. Compared to the staining intensity of ANGPTL7 in TALH cells of wild-type mice, TALH cells of *Umod* mutant mice exhibited a lower cytoplasmic staining intensity of ANGPTL7. Age of mice analyzed: four months. *Umod* ^wt^: wild-type mouse; *Umod* ^C93F^: homozygous *Umod* ^C93F^ mutant mouse. UMOD immunohistochemistry enabled identification of TALH segments. Serial kidney sections were used for ANGPTL7 and uromodulin immunohistochemistry and corresponding kidney regions are shown. C: renal cortex; P: renal papilla. Chromogen: DAB; nuclear staining: hemalum. (**B**) Protein abundance of ANGPTL7 in the outer medulla of kidneys of homozygous *Umod* mutant mice of both lines was decreased compared to wild-type mice. Signal intensities of ANGPTL7 were corrected for GAPDH signal intensities of the same PVDF-membrane. Mean of protein abundance of wild-type mice was set on a value of 1 [mean (wild-type)  = 1]. One-way-ANOVA with Tukey's Multiple Comparison Post hoc Test: *p* vs. wild-type, **, *p*<0.01; ***, p<0.001. Age of mice analyzed: four months.

In wild-type mice, ANGPTL7 protein was localized in the cytoplasm of all tubular cells, with more intense staining in TALH cells than in cells of other tubular segments (Figure 2A). In contrast to wild-type mice, the staining intensity of ANGPTL7 in TALH cells were less intense in *Umod* mutant mice, exhibiting a nearly similar staining intensity of ANGPTL7 in TALH cells like in cells of other tubular segments. Staining intensity of ANGPTL7 in non-TALH cells was similar irrespective of genotype. Identification of TALH segment was enabled by detection of UMOD. Western blot analyses of ANGPTL7 revealed a significantly decreased abundance of ANGPTL7 in the outer medulla of kidneys of *Umod* ^A227T^ and *Umod* ^C93F^ mutant mice compared to wild-type controls (Figure 2B).

**Figure 3:**
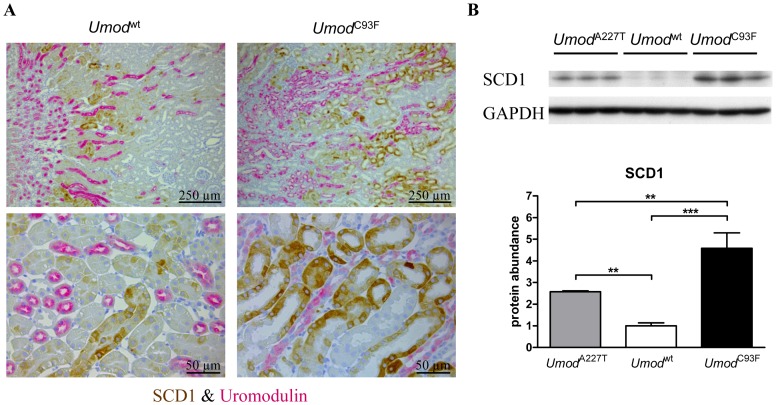
Analysis of localization and protein abundance of SCD1 in healthy and UAKD-affected kidneys. (**A**) SCD1 was detected in the cytoplasmic compartment selectively of proximal tubular cells (in the straight S3 segment). In the kidney of the homozygous *Umod* ^C93F^ mutant mouse, SCD1 appeared to be abundant in a larger fraction of proximal tubular cells compared to the kidney of the wild-type mouse, and the average staining intensity of SCD1 positive cells appeared to be more prominent. Age of mice analyzed: four months. *Umod* ^wt^: wild-type mouse; *Umod* ^C93F^: homozygous *Umod* ^C93F^ mutant mouse. Uromodulin immunohistochemistry enabled identification of TALH segments. Proximal tubule segment are morphologically characterized by luminal microvilli. Chromogen: DAB for SCD1, Vector RED for UMOD; nuclear staining: hemalum. (**B**) Protein abundance of SCD1 in whole kidney lysate of homozygous *Umod* mutant mice of both lines was increased compared to wild-type mice. Signal intensities of SCD1 were corrected for GAPDH signal intensities of the same PVDF-membrane. Mean of protein abundance of wild-type mice was set on a value of 1 [mean (wild-type)  = 1]. One-way-ANOVA with Tukey's Multiple Comparison Post hoc Test: *p* vs. wild-type, **, *p*<0.01; ***, p<0.001. Age of mice analyzed: four months.

SCD1 protein was localized in the cytoplasm selectively of proximal tubular cells of the kidney, concretely in the straight (S3) segment of proximal tubule (Figure 3A). Other tubule segments like TALH cells were negative for SCD1. In homozygous *Umod* mutant mice of both lines, SCD1 appeared to be abundant in a larger fraction of proximal tubular cells compared to wild-type mice, and the average staining intensity of SCD1 positive cells appeared to be more prominent. Western blot analyses of SCD1 revealed a 2.7-fold and 4.6-fold higher abundance in whole kidneys of *Umod* ^A227T^ and *Umod* ^C93F^ homozygotes compared to wild-type controls (Figure 3B).

### Evaluation of the role of TALH-dysfunction derived salt wasting and volume depletion state on Scd1 transcript abundance

**Figure 4:**
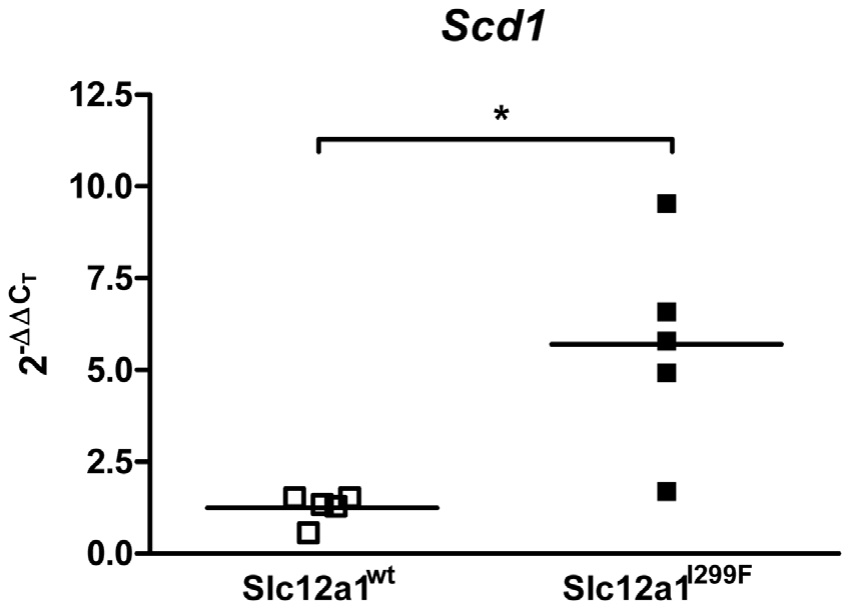
Evaluation of the role of TALH-dysfunction derived salt wasting state on renal *Scd1* transcript abundance. Increased *Scd1* transcript abundance in kidneys of homozygous *Slc12a1* ^I299T^ mutant mice compared to the kidneys of littermate controls were detected by RT-qPCR analysis. Data are shown as scatter dot plot with mean (n = 5 per group). Age of mice analyzed: three months. Student's t test: *p* vs. wild-type, *, *p*<0.05.

The bumetanide-sensitive ion transporter NKCC2 is mainly expressed in the kidney in the apical membrane of the cells of TALH and macula densa, and impaired function of NKCC2 due to inactivating mutations or due to the action of loop diuretics leads to salt wasting polyuria with reduced fractional excretion of uric acid [23]. Homozygous *Slc12a1* ^I229T^ mutant mice, suffering on TALH-dysfunction derived salt wasting and volume depletion state [14], exhibited a significantly increased *Scd1* transcript abundance in their kidneys compared to their littermate wild-type controls (Figure 4).

## Discussion

UAKD is a progressive hereditary disease and belongs to the endoplasmic reticulum (ER) storage diseases due to maturation defect of mutant UMOD and its retention in the enlarged ER of TALH cells [3]. Until know, little is known about the molecular alterations in UAKD affected kidneys, except of the induction of unfolded protein response and the recently identified activation of non-canonical NF-κB signaling in cells of the TALH segment [11]. In this study, we performed genome-wide transcriptome profiling of whole kidneys from young-adult homozygous *Umod* ^A227T^ and from aged heterozygous *Umod* ^C93F^ mutant mice exhibiting different severities of UAKD. With these analyses, we identified numerous DEGs in both mouse models of UAKD, which were functionally annotated with renal diseases and renal dysfunction. Further, numerous of the identified DEGs were found to be associated with the NF-κB signaling pathway. However, only 5 DEGs were identified being commonly differentially abundant in both *Umod* mutant mouse lines, but about 43% of the genes identified as DEGs in one of both *Umod* mutant mouse lines displayed a similar tendency of transcriptional alterations but being only significantly differentially abundant in one of the two mutant lines. This finding might be related to two facts of our study design:

First, in UAKD, primary functional and morphological alterations occur selectively in TALH cells, and TALH segments represent approximately 20% of whole kidney fraction. Using whole kidney for transcriptome profiling, this might be not suitable for detecting genes with altered abundances selectively in a small fraction of the kidney like in the TALH segment but being normally expressed also in other tubular segments as these molecular alterations in a small segment will be highly likely masked by the unaffected transcript abundance in the majority of kidney cells. However, our approach is highly suitable for identifying DEGs in UAKD expressed in a specific tubular fraction or whose transcript abundance is excessively altered in UAKD.

Second, the two groups of UAKD affected mice used for genome-wide transcriptome profiling exhibited different disease stages of UAKD. As recently shown, severity of UMOD maturation defect and speed of progression of UAKD *in vivo* strongly depends on the particular *Umod* mutation itself and the zygosity status [13]. The A227T mutation of *Umod* causes a milder UMOD maturation defect than the C93F mutation, and homozygosity of *Umod* mutation causes a stronger UMOD maturation defect and TALH dysfunction compared to heterozygous *Umod* mutant mice. Severity of UMOD maturation defect and severity of UAKD phenotype are similar between homozygous *Umod* ^A227T^ mutant mice and age-matched heterozygous *Umod* ^C93F^ mutant mice. UAKD is more progressed in 9-month-old heterozygous *Umod* ^C93F^ mutant mice than in 3-month-old homozygous *Umod* ^A227T^ mutant mice analyzed. Thus, the latter mice showed only an initial UAKD phenotype whereas the first group already exhibited progressed secondary morphological kidney lesions like interstitial fibrosis and inflammatory cell infiltrations. Consequently, on the one side, this sample selection of kidneys for genome-wide transcriptome profiling exhibiting different disease stages of UAKD might be responsible for the low number of identified genes commonly differentially abundant in both *Umod* mutant mouse lines. On the other side, these DEGs which were further positive validated by RT-qPCR of additional groups of UAKD-affected mice (*Angptl7*, *Odc1*, *Wfdc15b* and *Scd1*) might be the genes most constantly affected by UAKD irrespective of disease stage and severity. We selected two of the overlapping DEGs (*Angptl7* and *Scd1*) for more detailed analyses.

Angiopoietin-like protein 7 (ANGPTL7) was identified to be less abundant in TALH cells of UAKD affected kidneys. ANGPTL7 is a member of the ANGPTL protein family exhibiting structural homology to the angiopoietins, which have important functions in angiogenesis [24]. However the function of ANGPTLs might differ from angiopoietins as they do not bind to the receptors classically targeted by angiopoietins. So far, biological function of ANGPTL7, which is a secreted protein, is poorly understood. Due to its role in extracellular matrix formation of trabecular meshwork of the eye, a role of ANGPTL7 in glaucoma was assumed [25]. In this context, ANGPTL7 was postulated as potential target gene of the WNT/β-catenin signaling pathway [26]. Recently, ANGPTL7 was identified as a biomarker upregulated selectively in the early stage of acute kidney injury, with higher transcript abundances 4 to 10 hours after ischemia reperfusion injury (according to the data in patent #EP2582840A1, http://www.google.com/patents/EP2582840A1?cl=en). Further, strong upregulation of *Angptl7* transcription was detected in kidneys of hypertensive mice [27]. In UAKD, TALH cells are the site of primary pathogenesis due to mutant UMOD maturation retardation causing ER hyperplasia and TALH dysfunction. Decreased ANGPTL7 abundance in UAKD-affected TALH cells could be on the one side related to impaired synthesis due to impaired ER capacity and dysfunction. On the other side, as the biological function of ANGPTL7 in TALH cells is so far unknown, decreased ANGPTL7 synthesis might also contribute to TALH cell dysfunction leading to impaired kidney function in UAKD.

Increased abundance of stearoyl-coenzyme A desaturase 1 (SCD1) was present in proximal tubule segment of UAKD affected kidneys. SCD1 is a lipogenic enzyme catalyzing the critical step in the biosynthesis of monounsaturated fatty acids of cellular lipids [28]. The degree of unsaturation of cellular lipids is known to influence cell signaling and membrane fluidity and, thus, cell function. Due to its critical role in cell function, SCD1 expression is highly regulated. For instance, SCD1 expression can be induced by glucose, saturated fatty acids, insulin, and by the actions of lipogenic transcription factor sterol regulatory element binding protein-1c (SREBP-1c) and the nuclear receptor LXR. In the kidney, SCD1 was reported to be selectively expressed in the proximal straight tubular cells [29], which is in line with our results. It is an astonishing finding that disturbed TALH function as it is present in UAKD influenced transcript abundance of a gene selectively expressed in the tubule segment upstream of TALH. One key feature of UAKD represents a reduced renal fractional excretion of uric acid [3], and uric acid reabsorption and excretion is mainly regulated in the proximal tubule segment [30]. As a compensatory mechanism due to volume depletion caused by TALH dysfunction in UAKD, increased sodium absorption in proximal tubule, which is coupled with uric acid cotransport, was assumed in UAKD, leading to increased reabsorption and decreased excretion of uric acid [12]. Thus, higher SCD1 expression in proximal tubule of UAKD affected kidneys could be due to functional adaption of proximal tubule function to compensate TALH dysfunction for maintaining fluid and electrolyte homeostasis of the body. This hypothesis was underlined by our finding of increased *Scd1* abundance in kidneys of homozygous *Slc12a1* ^I299T^ mutant mice. These mice exhibited Type I Bartter syndrome with salt-wasting polyuria and reduced uric acid clearance due to an inactivating mutation of the Na^+^-K^+^-2Cl^—^ion transporter, which represents the main ion transporter of the TALH segment [14].

A direct cross talk between the two functionally distinct tubular segments, TALH and the morphologically contiguous S3 segment of proximal tubule, was assumed due to protective properties of UMOD, located in TALH segment, to acute injury damage in proximal tubules [31]. So, recovery of proximal tubule injury by ischemia was delayed in *Umod* knockout mice, which could be drawn back to increased inflammatory response in injured proximal tubule and increased neutrophil infiltration in *Umod* knockout mice compared to the ischemic injury kidney response in wild-type mice. A conceptual model of the UMOD dependent protective cross-talk of TALH segment on S3 segment of proximal tubule in acute kidney injury was proposed, postulating three putative paths of cross-talk between TALH and proximal tubule segment: (1) direct role of basolaterally translocated and released UMOD in down-regulation of inflammatory signaling in neighboring proximal tubule, (2) mediation of cross-talk by a putative secondary paracrine mediator released by TALH segment, or (3) involvement of interstitial cells in mediating cross-talk between the two functionally distinct tubular segments [32]. The increased abundance of SCD1 in straight segment of proximal tubule of UAKD-affected kidneys, as shown in our study, might be a further indication of the impact of TALH segment on the proximal tubule segment. The first proposed path of cross-talk due to reported translocated UMOD after ischemic injury might be of minor importance in UAKD-affected kidneys as we did not observe extracellular UMOD immunopositivity in renal interstitium. However, NF-κB signaling is activated in UAKD-affected TALH segment [11] what could be a putative mediator pathway of nephron segment cross-talk but this hypothesis has to be investigated in further analyses. Increased abundance of SCD1 itself might influence function of proximal tubule cells, by influencing their lipid metabolism and thus may alter the composition of phospholipids, triglycerides and cholesterol ester content in cell membrane [29]. Further, protective effects against lipotoxicity of free cholesterol and free saturated fatty acids, improvement of cell membrane fluidity and enhancement of cell function and integrity are assumed due to increased SCD1 abundance. Thus, the assumed increased sodium- and uric acid-cotransport in proximal tubule might be caused not only to compensate volume depletion in UAKD but also might be in part caused by altered cell function of proximal tubule cells itself.

In conclusion, transcriptome profiling analyses in whole kidneys of two *Umod* mutant mouse models for UAKD at two different stages of the disease resulted in the description of differentially regulated genes which were further classified according to disease and/or functions annotations. Localization and quantification analyses of ANGPTL7 and SCD1 gave novel hints for the function of these proteins in healthy and UAKD affected kidneys. A cross talk between two functionally distinct tubular segments, the TALH segment and the S3 segment of proximal tubule, was demonstrated, which might occur by direct contact or due to functional compensatory properties.

## Supporting Information

Figure S1 Clinical and morphological phenotype of young adult and aged *Umod* ^A227T^ and *Umod* ^C93F^ mutant mice. (A) Plasma urea concentrations of young adult and aged *Umod* ^A227T^ and *Umod* ^C93F^ mutant mice [13]. Data points show means ± SD. Age of clinical-chemical analysis is indicated. n = 6–16 per genotype and *Umod* mutant line. Two-way-ANOVA with Bonferroni Multiple Comparison Post hoc Test: Homozygous *Umod* mutants vs. wild type: ***, *p*<0.001; Heterozygous *Umod* mutants vs. wild type: ^###^, *p*<0.001. (B) Multifocal tubulointerstitial fibrosis and moderate inflammatory cell infiltration predominantly in the corticomedullary region were found in UAKD-affected kidneys of 12-month-old heterozygous *Umod* ^C93F^ mutant mice. These histological alterations were not found in three-month-old young adult homozygous *Umod* ^A227T^ mutant mice (not shown, [12]). Histological staining, age of mice and genotype are indicated. Het: heterozygous mutant, homo: homozygous mutant of the indicated *Umod* mutant mouse line.
(TIF)

Figure S2 Heat map of DEGs in kidney between homozygous *Umod^A227T^* mutant mice compared to wild-type controls. The genes are ranked according to their fold change from largest positive to largest negative value. The first column shows the mean linear fold changes calculated as ratio of the mean mutant intensities (n = 4) and the arithmetic mean signal of the corresponding wild-type mice (n = 4). Columns represent the regulated genes for single mutant mice and the rows the differential expression of a gene across the animals. The color code displays the fold changes according to the scale bar at the bottom of the heat map: Blue represents down- and yellow up-regulation in mutant animals compared to wild-types.
(XLS)

Figure S3 Heat map of DEGs in kidney between heterozygous *Umod^C93F^* mutant mice compared to wild-type controls. The genes are ranked according to their fold change from largest positive to largest negative value. The first column shows the mean linear fold changes calculated as ratio of the mean mutant intensities (n = 4) and the arithmetic mean signal of the corresponding wild-type mice (n = 4). Columns represent the regulated genes for single mutant mice and rows represent the differential expression of a gene across the animals. The color code displays the fold changes according to the scale bar at the bottom of the heat map: Blue represents down- and yellow up-regulation in mutant animals compared to wild-types.
(XLS)

Figure S4 Functional classification of DEGs in kidney of homozygous *Umod^A227T^* mutant mice. The first three columns list the categories of the diseases or functions annotations and the respective p-value for each over-represented term. The following columns show those DEGs associated with each term including a heat map and the mean fold change. The genes of each term are ranked according to the mean fold changes. A column represents single mutant mice and rows the differential expression across the animals. The color code displays the fold changes according to the scale bar at the bottom of the heat map: Blue represents down- and yellow up-regulation in mutant animals compared to wild-types.
(XLS)

Figure S5 Functional classification of DEGs in kidney of heterozygous *Umod^C93F^* mutant mice. The first three columns list the categories of the diseases or functions annotations and the respective p-value for each over-represented term. The following columns show those DEGs associated with each term including a heat map and the mean fold change. The genes of each term are ranked according to the mean fold changes. A column represents single mutant mice and rows the differential expression across the animals. The color code displays the fold changes according to the scale bar at the bottom of the heat map: Blue represents down- and yellow up-regulation in mutant animals compared to wild-types.
(XLS)
