# Supplementary figures and images for: Uromodulin Retention in Thick Ascending Limb of Henle's Loop Affects SCD1 in Neighboring Proximal Tubule: Renal Transcriptome Studies in Mouse Models of Uromodulin-Associated Kidney Disease
Source: PLoS One. 2014 Nov 19;9(11):e113125. doi: 10.1371/journal.pone.0113125 (PMC4237372; doi:10.1371/journal.pone.0113125)

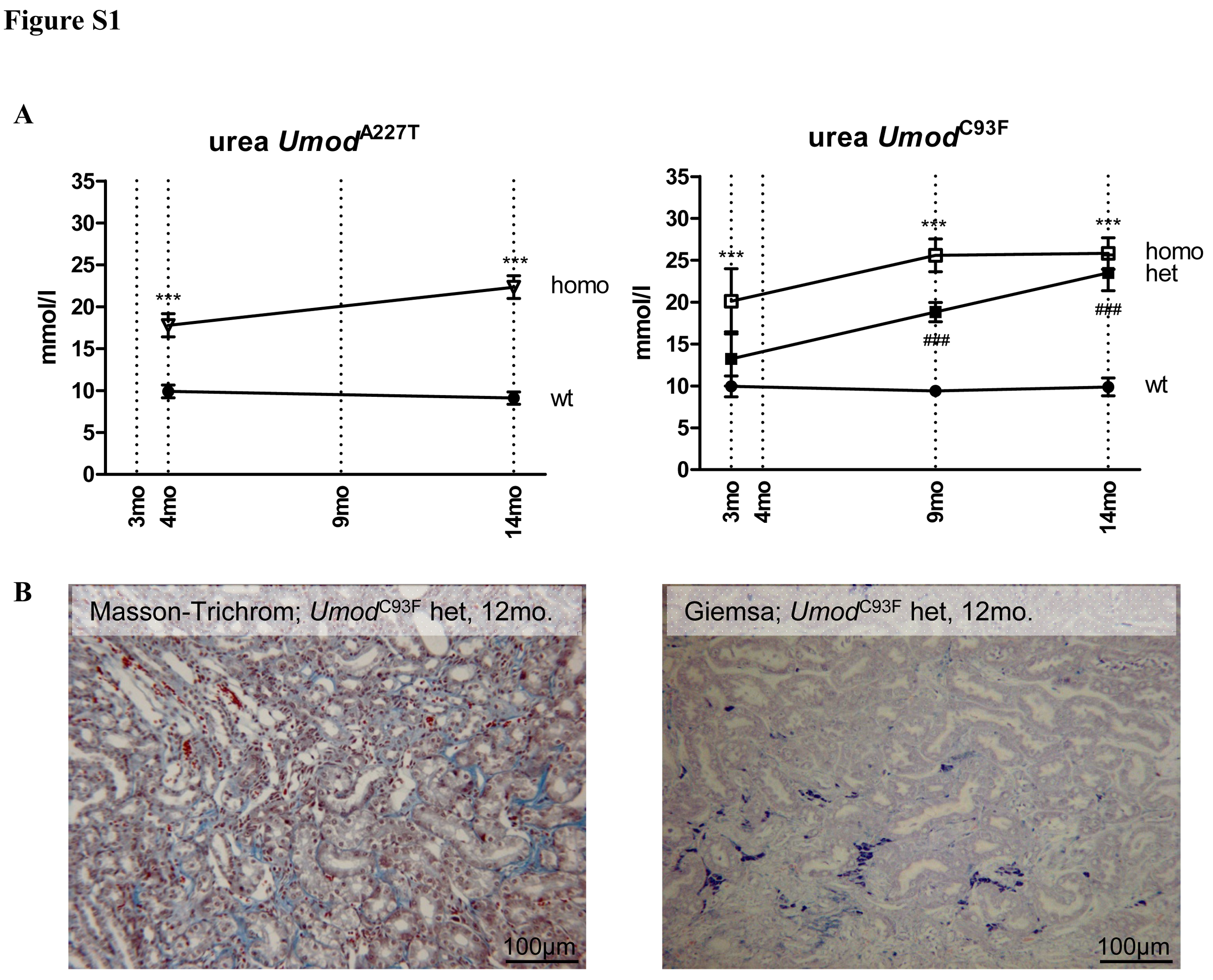

Supplement: Figure S1 — Clinical and morphological phenotype of young adult and aged Umod A227T and Umod C93F mutant mice. (A) Plasma urea concentrations of young adult and aged Umod A227T and Umod C93F mutant mice [13]. Data points show means ± SD. Age of clinical-chemical analysis is indicated. n = 6–16 per genotype and Umod mutant line. Two-way-ANOVA with Bonferroni Multiple Comparison Post hoc Test: Homozygous Umod mutants vs. wild type: ***, p<0.001; Heterozygous Umod mutants vs. wild type: ###, p<0.001. (B) Multifocal tubulointerstitial fibrosis and moderate inflammatory cell infiltration predominantly in the corticomedullary region were found in UAKD-affected kidneys of 12-month-old heterozygous Umod C93F mutant mice. These histological alterations were not found in three-month-old young adult homozygous Umod A227T mutant mice (not shown, [12]). Histological staining, age of mice and genotype are indicated. Het: heterozygous mutant, homo: homozygous mutant of the indicated Umod mutant mouse line. (TIF) [file pone.0113125.s001.tif]
